# An integrated whole genome analysis of *Mycobacterium tuberculosis* reveals insights into relationship between its genome, transcriptome and methylome

**DOI:** 10.1038/s41598-019-41692-2

**Published:** 2019-03-26

**Authors:** Paula J. Gomez-Gonzalez, Nuria Andreu, Jody E. Phelan, Paola Florez de Sessions, Judith R. Glynn, Amelia C. Crampin, Susana Campino, Philip D. Butcher, Martin L. Hibberd, Taane G. Clark

**Affiliations:** 1 Faculty of Infectious and Tropical Diseases, London School of Hygiene and Tropical Medicine, London, United Kingdom; 2 Genome Institute of Singapore, Biopolis, Singapore; 3 Faculty of Epidemiology and Population Health, London School of Hygiene and Tropical Medicine, London, United Kingdom; 4 Malawi Epidemiology and Intervention Research Unit, Lilongwe, Malawi; 5 Institute for Infection & Immunity, St George’s University of London, London, United Kingdom

## Abstract

Human tuberculosis disease (TB), caused by *Mycobacterium tuberculosis* (*Mtb)*, is a complex disease, with a spectrum of outcomes. Genomic, transcriptomic and methylation studies have revealed differences between *Mtb* lineages, likely to impact on transmission, virulence and drug resistance. However, so far no studies have integrated sequence-based genomic, transcriptomic and methylation characterisation across a common set of samples, which is critical to understand how DNA sequence and methylation affect RNA expression and, ultimately, *Mtb* pathogenesis. Here we perform such an integrated analysis across 22 *M. tuberculosis* clinical isolates, representing ancient (lineage 1) and modern (lineages 2 and 4) strains. The results confirm the presence of lineage-specific differential gene expression, linked to specific SNP-based expression quantitative trait loci: with 10 eQTLs involving SNPs in promoter regions or transcriptional start sites; and 12 involving potential functional impairment of transcriptional regulators. Methylation status was also found to have a role in transcription, with evidence of differential expression in 50 genes across lineage 4 samples. Lack of methylation was associated with three novel variants in *mamA*, likely to cause loss of function of this enzyme. Overall, our work shows the relationship of DNA sequence and methylation to RNA expression, and differences between ancient and modern lineages. Further studies are needed to verify the functional consequences of the identified mechanisms of gene expression regulation.

## Introduction

Human tuberculosis disease (TB), caused by *Mycobacterium tuberculosis* (*Mtb*), is a major global public health issue^1^. A deeper understanding of the biology of *Mtb* should reveal new insights that may help to improve diagnostics, treatments, vaccines and other much needed control measures. *Mtb* belongs to the *M. tuberculosis* complex (MTC), which consists of seven main lineages classified into modern (lineages 2–4), ancient (lineages 1, 5 and 6), and intermediate (lineage 7) strains^2^. The lineages vary in their geographic distribution and spread, with lineage 2 being particularly mobile with evidence of recent spread from Asia to Europe and Africa. Lineage 4 is common in Europe and southern Africa, coinciding with regions of high TB incidence and high levels of HIV co-infection. The lineages may vary in their propensity to transmit and to cause disease, and in the site and severity of disease^3–5^. A set of SNPs in the *Mtb* genome (size 4.4 Mb) has been identified that can be used to barcode sub-lineages^6^, leading to informatic tools that position sequenced samples within a global phylogeny^7^.

Genetic diversity, accessible through whole genome sequencing, plays an important role also in transcription. Gene expression differences have been observed, with 15% of the genes found to be differentially expressed among different *Mtb* clinical isolates^8^, and lineage-specific transcriptome differences have been observed *in vitro* and during survival in macrophages^9,10^. The mechanisms controlling expression of candidate genes, such as the upregulation of the *dosR* operon specific to Beijing strains, have been broadly investigated^11–13^. However, little is known about the effect of genomic variation on transcription at a whole genome scale. These effects can be explored through an association analysis of polymorphisms, such as single nucleotide polymorphisms (SNPs), and gene expression levels to determine expression quantitative trait loci (eQTL). eQTLs are genetic variants that explain variation in gene expression levels, and can be classified as *cis* or *trans* depending on the physical distance from the gene they regulate^14^. In *Mtb*, one previous study focusing on lineage 1 and 2 strains, highlighted two types of mechanisms where polymorphisms may change gene expression: through impairment of transcriptional regulators or by affecting the promoter regions^10^.

In addition to genomic variants, epigenetic mechanisms such as DNA methylation have an effect on gene expression. Several lines of evidence have revealed N6-methyladenine (m6A) and 5-methylcytosine (m5C) methylation mechanisms within *Mtb genomes*, and these can be characterised using single-molecule real time (SMRT) sequencing from Pacific Biosciences technology^15,16^. Motifs within three DNA methyltransferases (MTases), *mamA, mamB*, and *hsdM* are responsible for m6A modification^15–17^. In *Mtb* it has been shown that the loss of *mamA* MTase can decrease gene expression and affect survival during hypoxia^17^. Methylation sites have been found to overlap with sigma factor binding sites, suggesting that if methylation affects sigma factor binding, methylation status may play a role in transcription^17^. Lineage-specific methylation patterns have been reported for *Mtb* strains^16^, which indicates the potential for novel functional differences between them. In eukaryotic cells, DNA methylation is often associated with repression of gene expression; however, in prokaryotes, methylation has been associated with both induction and repression of gene expression^17,18^.

To date, no studies have integrated sequence-based genomic, transcriptomic and methylation characterisation across a common set of samples. This integration is critical to understand how DNA sequence and methylation affect RNA expression and, ultimately, *Mtb* pathogenesis. Here we seek to investigate the relationship between the genome, transcriptome and methylome in a panel of 22 *Mtb* isolates, belonging to the Karonga Prevention Study, a longitudinal epidemiological project focused on mycobacterial disease^19^. We present a differential gene expression study correlated with lineage, as well as an eQTL study linked with SNPs and methylated bases at a whole genome scale. Differential transcription between lineages was found, and genetic variants revealed as potential candidate eQTLs. Methylation status was also found to have a potential role in transcription, with evidence of differential gene expression between samples with non-methylated and methylated genes.

## Results

### Genomic analysis

**Figure 1:**
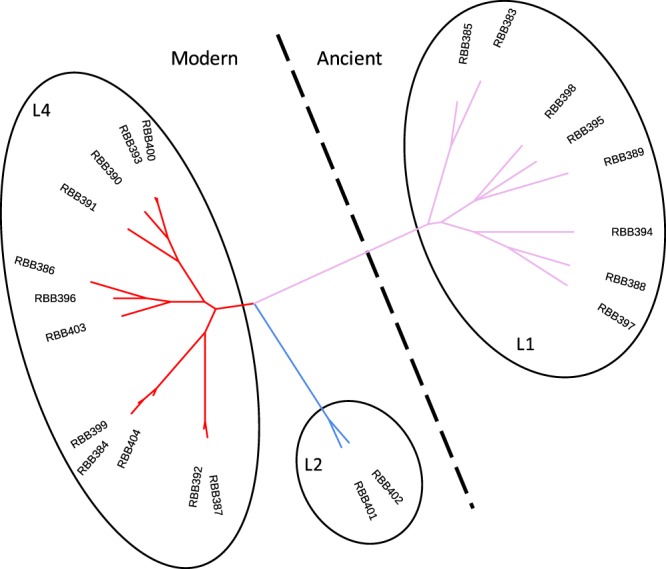
Phylogenetic tree of the 22 Karonga strains. Maximum-likelihood phylogenetic tree of the 22 isolates analysed, covering lineages 1 (L1), 2 (L2) and 4 (L4).

*Mtb* was isolated from 22 sputum samples from 22 different TB patients collected between 2003 and 2009 in Karonga, a northern district of Malawi. The majority of individuals were HIV positive (16/22). Genomic DNA was extracted and sequenced using PacBio single-molecule real time (SMRT) and Illumina sequencing technologies. One ancient (L1, n = 8) and two modern lineages (L2 and L4, n = 14) were represented (Supplementary Table S1). For each isolate, the raw sequence data was aligned to the H37Rv reference genome, leading to >100-fold average coverage. Across all samples 9,384 unique SNPs were characterised, with ~40% of them identified in single isolates. Only 1,446 of the 9,384 SNPs were located in intergenic regions. The average number of SNPs per isolate varied by lineage (L1: 2,613; L2: 1,675; L4: 1,101); the sub-lineage 4.9 (H37Rv-like) was the least polymorphic (~600 variants). Using the 9,384 SNPs, a maximum-likelihood phylogenetic tree was constructed (Fig. 1) and the isolates clustered by lineage as expected.

### Transcriptomic analysis and lineage-specific expression

**Figure 2:**
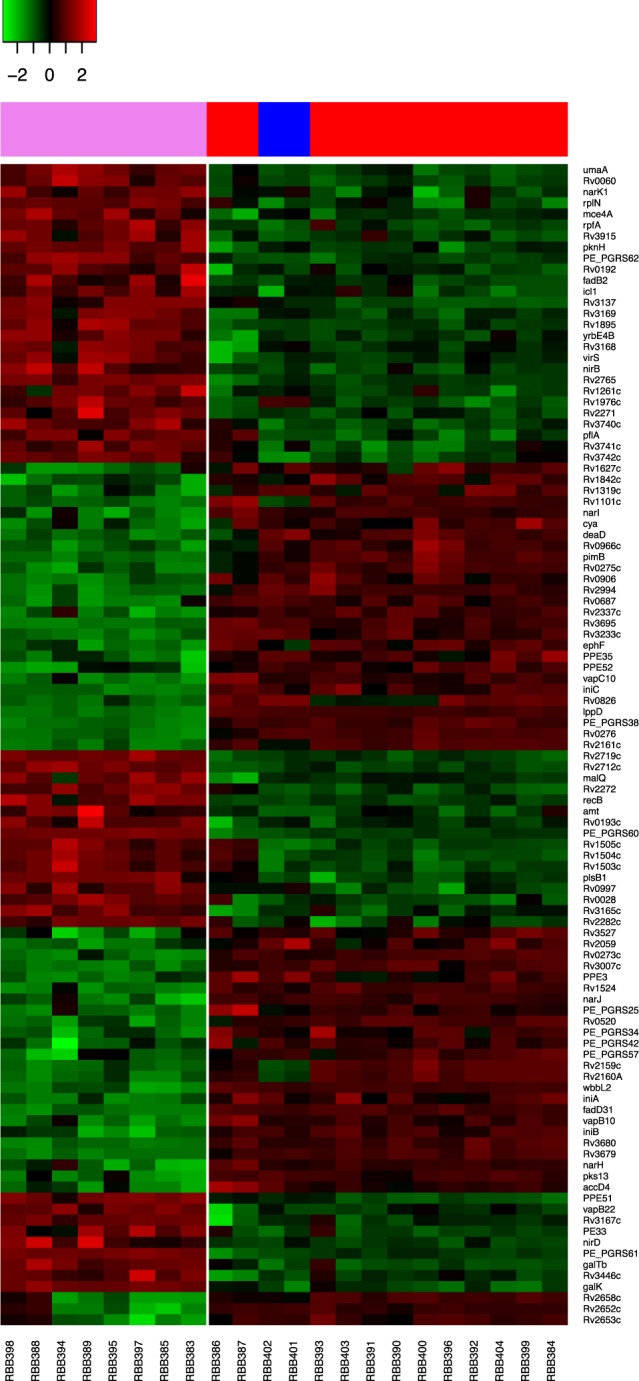
Gene expression differences between modern (lineage 2 and 4) and ancient (lineage 1) strains. A heatmap showing the 105 genes differentially expressed between ancient and modern strains, constructed with the gene expression distances between rows. Rows and columns are ordered based on row or column means. Over-expressed genes are coloured in red whilst under-expressed ones in green. Ancient strains (n = 8) represented on the left of the white vertical line and modern strains (n = 14) on the right. Lineage 1 represented in violet, Lineage 2 in blue and Lineage 4 in red.

*Mtb* RNA was extracted from the 22 clinical isolates following liquid culture at mid-log phase growth and sequenced using Illumina HiSeq technology. Short reads were aligned to the H37Rv reference genome and counts per gene were obtained. A total of 3,987 genes were transcribed in at least two clinical isolates with a minimum of 10 counts. The average number of transcripts in the sample set is 3,864. A differential expression test was performed by clade, between the ancient (L1; n = 8) and the modern (L2 and L4; n = 14) strains in our sample set (Supplementary Fig. S1A). At a significance level of *p* < 1.24 × 10^−5^ (corresponding to a Bonferroni adjusted *p* < 0.05), 105 genes were revealed as differentially expressed (Fig. 2, Supplementary Table S2). Five of them (*Rv1524*-*wbbL2*, *Rv2652c*-*Rv2653c*-*Rv2658c*) correspond to known deletions in ancient isolates. *PE_PGRS57* was also absent in ancient genomes of our samples, which has also been observed to be deleted in other ancient (L5; *M. Africanum*) strains in other studies^20,21^. As expected, *Rv1524*-*wbbL2*, *Rv2652c*-*Rv2653c*-*Rv2658c and PE_PGRS57* transcripts were down-regulated in ancient strains. Forty-eight of the 105 (45.7%) genes found to be differentially expressed by clade have been reported in previous transcriptomic analyses performed between ancient and modern strains or L1 and L2^9,10^, leading to 57 newly described genes here. The main functional ontological categories for the 105 identified genes were conserved hypotheticals and intermediary metabolism and respiration. Enrichment in nitrogen metabolism (*p* = 2.75 × 10^−5^) and PE-PGRS (*p* = 7.2 × 10^−3^) associated genes was found. Within clade-specific patterns, genes associated with transcriptional regulation were also identified. For ancient strains, *Rv0273c*, *Rv0275c*, and *Rv2160A* were the most under-expressed, whilst *pknH*, *Rv2282c*, *virS*, and *Rv3167c*, were over-expressed. In addition, several of the 105 differentially expressed genes were associated with virulence. Three of them belonged to the *vapBC* toxin-antitoxin system (*vapB10*, *vapC10*, *vapB22*), which were up- or down-regulated in ancient strains. Also, the *mce4A* gene, involved in cholesterol uptake during macrophage survival and associated with long term persistence^22^, and *yrbE4B*, forming part of the *mce4* operon, were found over-expressed in ancient isolates. Finally, genes associated with drug resistance, such as the efflux pump *Rv2994* and the isoniazid related *iniA* and *iniB* genes, were revealed as differentially expressed between the ancient and modern lineages studied. *Rv2994* has found to be over-expressed in multi-drug resistant isolates^23^, and the *iniA* and *iniB* genes are related with higher persistence under isoniazid conditions^24,25^.

### Identification of Expression Quantitative Trait Loci (eQTL)

An eQTL analysis was performed at a whole genome scale across the 22 isolates, and we attempted to associate SNP alleles with differential transcription signal. Association testing was performed between 9,384 SNPs and 3,987 transcripts using a linear regression modelling approach (Supplementary Fig. S1B). We identified potential eQTLs from the 38,949 significant associations between 5,608 SNP positions and 118 differential transcribed genes (*p* < 1.32 × 10^−9^; adjusted *p* < 0.05). The 5,608 SNPs considered as eQTLs were located in 2,279 genes and intergenic regions. Forty-two of the 118 (35.6%) genes were differentially expressed due to large deletions and were subsequently excluded from further analysis **(**Supplementary Table S3), leaving 76 genes as potentially affected by SNP eQTLs (Supplementary Table S4). More than half of these 76 genes had a lineage or sub-lineage-specific expression profile. Moreover, a large number of the eQTLs associations were due to both lineage-specific SNPs and expressed genes. Thereby, a group of 790 common SNPs across all ancient isolates was associated with the expression of 24 genes; a group of 169 SNPs present in all L1 and L2 isolates was associated with the expression of 9 genes, and 584 SNPs present in Beijing (L2) isolates were associated with the expression of 3 genes (Supplementary Table S4). To assign the most likely causative genetic variation of the eQTLs, we investigated SNPs with a potential *cis* regulatory function and those within transcriptional regulatory proteins.

#### Cis-regulatory eQTLs

A *cis*-eQTL analysis was performed at SNPs, within each gene or < 200 bp upstream from their start codon, tested for differential expression (Supplementary Fig. S1C). This analysis identified 99 potential *cis*-eQTLs associated with the differential expression of 83 genes (*p* < 4.04 × 10^−6^, adjusted *p* < 0.05), involving 92 SNPs (Supplementary Table S5). The majority (65/92) of these candidate *cis*-eQTL SNPs were located within the gene, 15 were located in the upstream intergenic region and 8 within the upstream gene. Among those in the upstream intergenic region, 8 were in predicted promoter regions. Eleven upstream SNPs (11/15) were common (allele frequency >5%) in a global set of strains (n = 6,218)^26^. Also, 6 SNPs within the upstream gene (6/8) were common (Table 1). Among them, the antitoxin *vapB22*, is known to be over-expressed in ancient isolates when compared to modern strains, and was found to harbour a SNP in its promoter (T3137237C) in all ancient isolates, thereby providing a possible explanation for the change in expression. Further, all the SNPs identified as potential *cis*-eQTLs were aligned to a map of transcriptional start sites (TSS)^27^. We found that three were located within the TSS of three genes shown to be differentially expressed in L1 compared to modern strains, with *PE_PGRS38* (A2424864G) and *fadD31* (T2177073C) under-expressed, and *virS* (A3447480C) over-expressed in ancient isolates. Overall, five SNPs present in ancient strains identified in this study as potential *cis*-eQTLs have already been reported as potentially associated with variation in gene transcription^10^, giving us confidence in our approach.

**Table 1:** Putative functional SNPs associated with expression (*cis-*eQTLs with allele frequencies >5%; adjusted *p* < 0.05).

	Transcript differentially expressed	Annotation	SNP	Position SNP			Regulation	Strain Lineage	Allele frequency**	
				Gene	Distance (bp) from start codon	Promoter (P)/TSS			Ancient	Modern
SNPs in upstream region	*Rv0193c*	1	G226676A	IGR	−105	—	Up	1	0.973	0
	*Rv0326*	—	T392261C	*Rv0325*	−12	—	Up	1,2	0.978	0.324
	*Rv0377*	6	T454295C	*Rv0376c*	−126	—	Up	1,2,4.1,4.3.4, 4.8,4.9	1	0.994
	*gpdA1*	4	T655986G	IGR	−37	P	Up	1,2	0.976	0.324
	*mce2D*	6	A690450C	*mce2C*	−51	—	Up	1,2	0.976	0.324
	*Rv0669c*	3	T769663G	IGR	−66	P	Down	4.3.3	0	0.050
	*Rv0958*	3	C1069871T	IGR	−12	P	Up	1.1.3	0.220	0
	*Rv1096*	3	T1224367C	IGR	−18	P	Down	1,2,4.1,4.3,4.8	1	0.976
	*Rv1503c*	1	A1694547C	IGR	−3	—	Up	1	0.973	0
	*fadD31*	4	T2177073C	IGR	−14	TSS/P	Down	1	0.973	0
	*Rv2036*	3	C2282058T	*Rv2035*	−41	—	Up	1.2.2*	0.157	0
	*Rv2159c*	1	A2421816G	*Rv2160A*	−151	—	Down	1,2	0.977	0.323
	*PE_PGRS38*	7	A2424864G	IGR	−18	TSS	Down	1	0.973	0
	*Rv2712c*	1	C3025431T	IGR	−103	P	Up	1	0.971	0
	*vapB22*	5	T3137237C	IGR	−13	P	Up	1	0.973	0
	*yrbE4B*	5	G3920109T	*yrbE4A*	−47	—	Up	1	0.971	0
	*Rv3695*	2	T4137190C	IGR	−16	—	Down	1	0.973	0

Table showing the candidate transcripts differentially expressed due to SNPs in upstream intergenic regions (IGRs) or within the upstream gene. Annotation of the transcript differentially expressed: 1 – Conserved hypotheticals, 2 – Cell wall and cell processes, 3 – Intermediary metabolism and respiration, 4 – Lipid metabolism, 5 – Virulence, detoxification, adaptation, 6 – Regulatory proteins, 7 – PE/PPE, 8 – information pathways. Distance of the SNP location from the start codon of the transcript is showed as negative when it is upstream and positive when it is located within the gene. TSS = Transcriptional Start Site. *Only one or two samples from the lineage out of the 3 analysed. **Allele frequency refers to the fraction of strains harbouring the SNP in a larger data set (n = 6,218)^50^; “—“ when not available.

#### Transcriptional regulatory proteins

We next considered candidate SNP eQTLs with non-synonymous mutations in transcriptional regulatory proteins (Supplementary Fig. S1D). These mutations could affect the DNA binding function of the protein. In total, 46 SNPs in 38 different transcriptional regulatory proteins (Table 2) were associated in the eQTL analysis with the differential transcription of 56 genes, accounting for a total of 376 potential eQTL associations. Ten of these 46 SNPs have been previously reported as having a potential effect in transcriptional regulation^10^. Functional effects were investigated through the SIFT algorithm, and 16 of the 38 (42.1%) transcriptional regulators were predicted to have SNP mutations affecting functional impairment. For the majority of the regulatory genes (20/38; 52.6%), the SIFT software did not predict a functional consequence of the mutations, due to the lack of homology with sequences in its database.

**Table 2:** Non-synonymous variants in transcriptional regulatory genes with eQTL associations, with potential functional impairment.

Gene	Mutation	Family	Lineage of strains carrying mutation	Allele frequency	
				Ancient	Modern
*whiB5*	S21G	whiB	1.2.2**	0.021	0
*Rv0023*	G217D		4.9**	0	0.001
*Rv0042c*	L186R*	MarR	4.9**	0	0
*Rv0144*	P36L*	tetR	4.9**	0	0
*Rv0195*	C41STOP	LuxR	1.2.2**	0.021	0
*Rv0275c*	S24L	tetR	1	0.973	0
*iniR*	E23K		1.2.2**	0.019	0
*Rv0377*	P302R*	LysR	1	0.973	0
*Rv0386*	L475R*	LuxR/UhpA	4.1.1.3	0	0.003
*ramB*	P91Q		1	0.973	0
	T118A		4.9**	0	0.001
	Q121R		1	0.973	0
*Rv0576*	R233H*	ArsR	1,2	0.978	0.334
*Rv0691c*	A140T		2	0.003	0.114
*Rv0818*	P227L*		4.1.1.3	0	0.003
	E246K*		4.1.2	0	0.009
*narL*	G169R*		2	0.003	0.147
*Rv0890c*	E234G*	LuxR	2	0.003	0.111
	E303K*		4.1.2	0	0.009
*Rv0891c*	V37G*		1,2,4.1,4.3,4.8	1	0.974
*kdpE*	G60S*	KDPD/KDPE	2	0.003	0.111
*Rv1219c*	R11T		1.2.2**	0.148	0
*embR*	A70S		4.1.2	0	0.009
	C110Y		1	0.973	0
*Rv1453*	D208N		1.1.3	0.230	0
	D218N		1.2.2**	0.021	0
	P405Q		1,2,4.1,4.3,4.8	1	0.974
*Rv1674c*	E189G*		4.3	0.014	0.281
*cmr*	V59A	CRP/FNR	1	0.974	0
	A125S		1.1.3*	0.072	0
*Rv1776c*	R154S		1.2.2**	0.019	0
*blaI*	L57R		1	0.970	0
*mce3R*	D148Y*	tetR	1.1.3**	—	—
*Rv2017*	A262E		1,2,4.1,4.3,4.8	0.998	0.973
*Rv2160A*	C155R	tetR	1,2	0.977	0.323
*zur*	H64R*		1	0.973	0
*Rv2488c*	D184Y*	LuxR	1.2.2**	0.018	0
*Rv2621c*	A110V		2	0.003	0.148
*sirR*	Q131STOP		1	0.973	0
*Rv3060c*	G420D	GntR	4.1.2	0	0.009
*virS*	L316R*	AraC/XylS	1	0.973	0
*Rv3167c*	P17Q	tetR	1	0.973	0
*Rv3249c*	T154A	tetR	4.1.1.3	0.003	0.049
*whiB4*	S2L	whiB	1.1.3	0.223	0
*Rv3736*	G144R*	AraC/XylS	1	0.971	0
*whiB6*	G71D	whiB	1.2.2**	0.014	0

Table showing non-synonymous mutations in transcriptional regulatory genes found as potential eQTLs. *Sorting Intolerant from tolerant (SIFT) predicted scores (*p* value) < 0.05 and considered to have functional impact; whilst for the others the SIFT software was unable to predict functional effects of mutations; **Only one or two samples available from the lineage. Allele frequency refers to the fraction of strains harbouring the SNP in a larger data set (n = 6,218)^26^.

Mutations in the *sirR* and *Rv0195* genes resulted in stop codons and led to truncated proteins. The stop codon in *sirR*, a manganese-dependent transcriptional repressor^28^, was observed in all L1 samples. While, mutations in *Rv0195*, a LuxR family regulatory gene, were observed in one L1 sample. Some of the 38 transcriptional regulators belonged to other known regulatory families such as TetR. The TetR family of transcriptional regulators (TFTRs) are one-component prokaryotic signal transduction systems controlling different biochemical functions. Although they were thought to be expression repressors, work in other bacteria has shown that they can act also as activators^29^. The TFTR *Rv2160A* carried a SNP (C155R) and an insertion (304insGGAA) causing a change in the reading frame in isolates from L1 and L2. *Rv2160A* is likely to form part of the operon *Rv2159c*/*Rv2160A*/*Rv2161c*. In our analysis, *Rv2159c* and *Rv2161c* were revealed as highly down-regulated in ancient strains compared to modern ones, and marginally down-regulated in L2 compared to L4 isolates. These observations suggest the operon may act as an activator, and that the mutations may lead to a loss of its function.

In *Streptomyces* it has been shown that TFTRs can regulate divergently oriented neighbouring genes^30^, and previous studies in *Mtb*^10,31^ have found differential expression of genes adjacent to TFTRs. We looked for similar effects in *Mtb* TFTRs carrying potential eQTLs. *Rv0275c* is a potential regulator of its divergent oriented neighbouring gene *Rv0276*. The ancient strains carried a mutation (S24L) in *Rv0275c*, which was associated with the under-expression of *Rv0276*. Similarly, *Rv3167c* is a potential regulator of its divergent oriented neighbour gene *Rv3168*. Although, the ancient strains carried a mutation (P17Q) in *Rv3167c,* and *Rv3168* appeared slightly over-expressed, this effect did not reach the stringent significance cut off imposed in the eQTL analysis.

In order to study the consequential effects of mutations in the transcriptional regulators of the genes found as being differentially expressed, network gene regulation was analysed through the Environment and Gene Regulatory Influence Network (EGRIN) model from the MTB Network Portal^32^ and the regulatory network map from the TB database^33^. We compared the predicted induced and repressed genes by the transcriptional regulators harbouring non-synonymous SNPs with the differentially expressed genes in our samples. This analysis revealed the association of genes differentially expressed with five of our candidate transcriptional regulators (Supplementary Table S6). *Rv0275c*, which is predicted to auto-induce its expression, was found to be down-regulated in ancient strains (with S24L mutation), although this effect did not reach the statistical significance cut-off. In addition to the under-expression of *Rv0276*, discussed above, three other genes (*Rv0520*, *Rv2162c* and *Rv0826*) were found to be under-expressed in ancient strains and are predicted to be regulated by *Rv0275c*. Genes regulated by *ramB*, were up- or down-regulated in ancient strains carrying *ramB* P91Q and Q121R mutations. Other genes were regulated by the transcriptional regulators *Rv1776c*, *Rv3167c* and *Rv3249c*, which harboured potential impairment mutations, leading to under- or over-expression in those isolates carrying the mutations. For the remaining regulators within known control networks, no statistically significant associations of variable gene expression with mutations were found.

Sigma and anti-sigma factors are critical to the gene expression regulatory network^34^, and here we hypothesised that polymorphisms in these factors might affect the transcription of those genes regulated by them. We found three anti-sigma factors (*rseA, rskA* and *rsfA*) harbouring non-synonymous SNPs that were considered as potential eQTLs (adjusted *p* < 0.05) associated with six genes differentially expressed between the isolates carrying and not carrying the mutations (Supplementary Table S7).

### Methylation analysis

Motif and methylation finding was performed through the Modification and Motif Analysis pipeline provided by the SMRT portal (https://github.com/PacificBiosciences/SMRT-Analysis). By analysing the kinetic variation through the inter-pulse duration ratio (IPD) at each nucleotide in the genome, a large number of modifications were identified. Only high quality 6-methyl-adenine (m6A) levels were found within motifs, where m6A is a well characterised epigenetic regulator in other prokaryotes^35,36^. The three motifs previously reported in *Mtb*^15–17^ were identified: CTCCAG and GATN_4_RTAC and their partner motifs (CTGGAG and GTAYN_4_ATC, respectively), and the hemi-methylated CACGCAG. The distribution and numbers of the different motifs were similar across the samples regardless of lineage and sub-lineage, with an average number of 1,934 for CTCCAG, 357 for GATN_4_RTAC and 813 for CACGCAG. However, the fraction of methylated motifs varied across isolates and (sub-)lineage patterns (Supplementary Table S8), consistent with a previous report^16^. In particular, within L4, two sub-lineages patterns were found with methylation in GATN_4_RTAC and CACGCAG motifs. Moreover, the CTCCAG motif was not methylated in either of the two L2 isolates. Among L1, methylation in CTCCAG and CACGCAG motifs was absent in some samples. When methylated, the percentage of motifs modified across all the samples varied from 50% to ~100%.

To explain the lack of methylation observed in some isolates, the presence of SNPs in the MTases genes was investigated. Three SNP mutations were identified: (i) E270A in *mamA* in L2, (ii) P306L in *hsdM* in sub-lineages 4.3, 4.8 and 4.9, and (iii) S253L in *mamB* in sub-lineage 1.1.3; which have been reported previously to be associated with the loss of function of the enzymes^15,16^ (Supplementary Table S9). Two novel mutations (Q340K and G152S) and a deletion (1232delG) were also identified in *mamA*, potentially associated with the lack of methylation of CTCCAG in two isolates belonging to L1 and L4. For the remaining samples with an absence of methylation in any of the three motifs, there were no SNPs uniquely found in these samples that could be correlated with the loss of function of the enzyme.

### Differential gene expression linked with methylation

In order to understand how the methylation status of the genes affects their expression, a differential transcription analysis was performed on the L1 and L4 strains (n = 20) (Supplementary Fig. S1E). The analysis involved stratifying by lineage to overcome the lineage-specific transcriptional profiles seen above. L2 was discarded due to the low number of clinical isolates represented. Firstly, 5,326 different intragenic methylation sites were used. A linear regression analysis was applied to obtain the correlation between methylation status and gene expression level at a whole-genome scale. Across L4, 44 genes were found to be differentially expressed (Benjamini-Hochberg (BH) adjusted *p* < 0.05), whose over- or under-expression was potentially associated with their methylation status. Twenty-eight (of the 44; 63.6%) genes, mostly down-regulated, were deficient in methylation only in the CTCCAG motif in one sample, which was associated with the presence of the mutation G152S in *mamA* (Supplementary Fig. S2). These genes were enriched for metabolic pathways (*p* < 0.05). The remaining 16 genes differentially expressed in L4 were non-methylated in >1 isolate and mostly in the CTCCAG motif (Supplementary Fig. S3). For L1, none of the genes that were found to be differentially expressed were significantly associated with methylation status. Methylation of the upstream intergenic regions may have a role in gene expression, and we performed a lineage-stratified *cis*-eQTL analysis with the 393 unique methylation sites located within 200 bp upstream from the start codons of the genes. In L4, seven eQTLs (BH adjusted *p* < 0.05) for 8 genes differentially expressed were revealed (Table 3, Supplementary Fig. S4), including one located in the predicted promoter region and overlapping with the TSS. Among ancient strains, none of the genes that were found to be differentially expressed were significantly associated with methylation of upstream regions.

**Table 3:** *cis*-eQTLs located in upstream intergenic regions linked with methylation in Lineage 4 strains.

Gene	Position	strand	Motif	Distance from start codon (bp)	Promoter*/TSS*	Regulation in non-methylated samples
*Rv0565c*	657533	−	CTGGAG	−63	−	Down
*ompA*	1002711	+	CTCCAG	−101	−	Down
*Rv1371*	1543277	+	CTCCAG	−82	−	Up
*scpB*	1938088	+	CTCCAG	−58	P, TSS	Up
*moaC3*	3710411	−	CTCCAG	−163	−	Up
*Rv3324A*	3710411	−	CTCCAG	−32	−	Up
*Rv3325*	3710408	+	CTGGAG	−25	−	Down
*PE_PGRS60*	4093563	+	CTGGAG	−69	−	Down

Table showing genes differentially expressed potentially due to the lack of methylation in the upstream region. The name of the gene, the position of the eQTL (methylation site), strand, motif, distance of the methylated base from start codon of the transcript (negative shown as upstream), prediction of promoter or TSS (P = promoter region, TSS = Transcriptional Start Site), and type of regulation of the gene in non-methylated samples is shown.

### Overlap between eQTLs linked with SNPs and methylation

**Figure 3:**
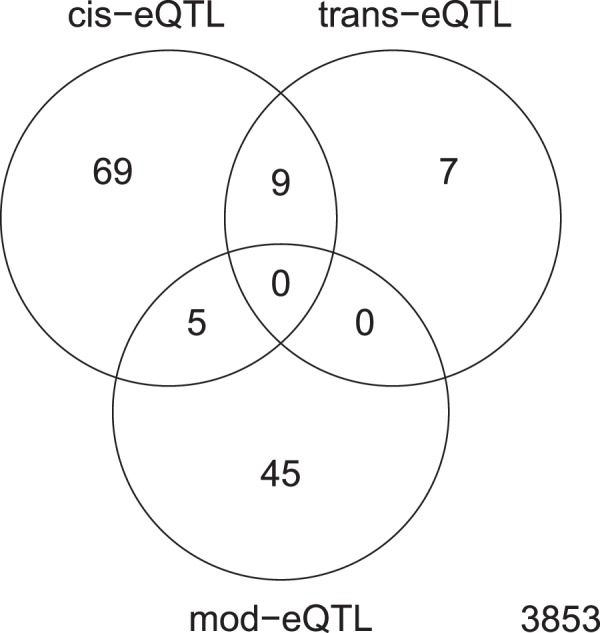
Venn diagram showing the overlap of genes differentially expressed (from the 3,987 investigated) associated with the different eQTL types (*cis*, *trans* and modified). The numbers represent the number of genes differentially expressed associated with the different types of eQTLs: *cis*-eQTLs, SNPs in promoter regions, transcriptional start sites (TSS), upstream (up to −200 bp) or within the gene; *tr*-eQTLs, potentially impairing non-synonymous SNPs located in transcriptional regulators; and *mod*-eQTLs, methylated bases located either within the gene or upstream including promoter regions and TSS.

Finally, we assessed whether there is a link between the SNPs and methylated motifs associated with the differentially expressed genes identified. To this end, we evaluated the degree of overlap between the different associations (Fig. 3). We considered three types of association: (i) genes differentially expressed due to SNPs in promoter regions, TSS or within the gene, denoted as *cis*-eQTLs; (ii) genes differentially expressed due to potential impairing mutations in transcriptional regulators that are predicted to control their expression, denoted as *tr*-eQTLs; and (iii) genes differentially expressed as a consequence of methylation of either the promoter, TSS, upstream region or the gene, denoted as *mod*-eQTLs. We found that 5 genes with variable transcription were associated with both, *mod*-eQTLs and *cis*-eQTLs, and another 9 were associated with *cis*-eQTLs and *tr*-eQTLs. There was no overlap between genes differentially expressed due to *tr*-eQTLs and *mod*-eQTLs, and the majority of the genes were uniquely assigned to one of the mechanisms responsible for their differential expression.

## Discussion

Genetic mutations and variations in gene expression have an important impact on MTC virulence and pathogenicity^4,5^. Previous studies have shown how genomic variants or methylation can affect the level of gene expression^9,10,17^, but have not shown how one analysis may influence another. In this study, for the first time, we performed an integrated analysis of the genome, methylome and transcriptome, across 3 major *Mtb* lineages. We have revealed clade-specific differences in the core transcriptomes between ancient and modern strains, as previously observed^9^, but in addition our analysis has revealed genes linked to virulence and pathogenicity (e.g. *vapBC* family), drug resistance and efflux pumps (e.g. *Rv2994*^23^ or *iniA* and *iniB*^24,25^). An eQTL analytical approach revealed 5,608 SNPs associated with differential gene expression (a total of 38,949 candidate eQTLs) and reinforced the lineage-specific genetic diversity and its effects on transcriptomes. To achieve improved resolution, *cis*-eQTLs based on regions upstream or within the genes differentially expressed were considered. This approach revealed ten SNPs within the promoter regions or TSS of genes differentially expressed, as well as others within coding regions of the genes, doubling the number of previously reported associations^10^. Among these variants, lineage-specific SNPs were associated with the genes differentially expressed, thereby revealing a potential explanation for the differential core transcription.

The high proportion of non-synonymous mutations present in coding regions in *Mtb* has been suggested to have a functional impact^4^, with consequences for transcription when found within transcriptional regulators^10^. In our study, functional impairment was predicted for sixteen of the transcriptional regulators found among the 38,949 potential eQTLs, including in *sirR* and *Rv0195* that contained premature stop codons. The number of regulators found is likely to be an under-estimate, as databases accessible to SIFT are incomplete, leading to no prediction for the vast majority of loci. Most of the potential impairing mutations were found to be lineage-specific. In particular, we identified a mutation and an insertion in L1 and L2 strains in *Rv2160A*, which act as a transcriptional activator of the adjacent genes *Rv2159c* and *Rv2161c*, with which it likely forms an operon^29^. Similarly, the protein encoded by *Rv3167c* was predicted to function as a repressor of its contiguous gene *Rv3168*, over-expressed in ancient samples with the P17Q mutation. Whilst *Rv0275c* was shown as a candidate activator of the adjacent gene *Rv0276*, and under-expressed in the L1 strains with the S24L mutation, consistent with previously reported associations^10,31^. The analysis of the regulatory networks of the transcriptional regulators was performed in order to look for *trans*-eQTLs, and found 11 of the genes differentially expressed from the primary eQTL analysis were regulated by one of the transcriptional regulators harbouring potential impairing mutations. Three mutations affecting the function of three anti-sigma factors (*rseA, rskA* and *rsfA*) were associated with the up-regulation of 6 genes. This result suggests that the functional impairment of sigma and anti-sigma factors can be the cause of variable gene expression.

Our study confirmed the same motifs and patterns of methylation as previously reported^15,16^ but in addition identified three novel variants (Q340K, 121delG and G152S) in *mamA*, which could explain the lack of methylation in the CTCCAG motif in the samples harbouring them. DNA methylation has been hypothesised to affect gene expression in bacteria^35^, and the disruption of *mamA* in *Mtb* has been shown to result in altered gene expression^17^. In *E. coli* it has been suggested that an overrepresented motif in the genome is more likely to be involved in gene expression regulation mediated by methylation^37^. Different hypotheses concerning the control of gene expression by *dam* MTase have been proposed, including regulation by motifs found in promoter^38^ and coding regions^39^. Further, it has been suggested that DNA methylation is a mechanism of switching regulatory states in phase variation systems^37^. Across the three lineages studied here, CTCCAG was the most abundant motif and was predominantly found in coding regions. An investigation of the relationship between the methylation status and gene expression levels revealed that the CTCCAG motif has the highest impact. In L4, the differential expression of 38 genes was potentially associated with CTCCAG methylation status, compared to 4 and 2 genes associated with CACGCAG and GATN_4_RTAC methylation, respectively. A subset of these genes (28/44), mostly down-regulated, were found to be uniquely non-methylated in the sample with the *mamA* G152S mutation. These included genes associated with metabolic pathways or regulatory proteins (e.g. *Rv0348*, *virS* or *Rv1359*), and from the *pe/ppe* families (e.g. *PE17, PPE17* or *PE_PGRS2*). We also found that non-methylated CTCCAG motifs in upstream regions and TSS have an effect on gene expression, which is consistent with previous work^17^. In L1 no genes significantly associated with methylation were found. Overall our results show that methylation in the promoter regions and coding regions is likely to be involved in gene expression, with the CTCCAG motif as the main candidate with a role in regulation.

The functional impairment of MTases may have implications in biological processes of the *Mtb* controlled by genes whose expression is affected by the methylation status. This could eventually influence the *Mtb*’s virulence, pathogenicity or drug resistance. For instance, variable methylation status was found to be related to the differential transcription of genes associated with metabolic pathways, among others, which suggests the potential role of methylation on regulation of biological processes related with growth or persistence. However, further work is needed to understand how methylation regulates gene expression under different environmental cues including those encountered by *Mtb* inside the host.

In *Mtb*, virulence and the ability to become drug resistant vary across lineages^40,41^. Hence, the study of lineage-specific transcriptomic profiles and the mechanisms that regulate gene expression can give insights into mechanisms underlying these biological differences. Such insights will be useful to identify potential targets for the development of new anti-tuberculosis drugs or vaccines. The small sample size is a potential limitation of the study, but our integrated analysis has detected known variants and methylated motifs, and putative candidate eQTLs for follow-up experiments. Future studies should consider larger sample sizes, including more lineages (e.g. other ancient lineages, such as L5 and L6), in order to confirm the candidate associations found in this analysis. In addition, there is a need for complementary proteomic analyses, to perform a comprehensive integrated study of *Mtb* genetic and epigenetic mechanisms of gene expression control. Overall, our data has identified common functional variants that affect transcriptional control, which gives further support to differential pathophysiology in ancient and modern *Mtb* lineages.

## Materials and Methods

### Bacterial strains, DNA and RNA sequencing

All 22 *Mtb* isolates listed in Supplementary Table S1 were sourced from 22 TB patients from Karonga (Malawi) between 2003 and 2009, and cultured in the LSHTM. *Mtb* isolates were grown by liquid culture (in the absence of antimicrobial drugs) from frozen stocks of Lowenstein-Jensen or liquid cultures derived from patient’s sputum specimens already isolated. *Mtb* strains were grown to mid-log phase (OD = 0.6–0.8) in Middlebrook 7H9 supplemented with 0.05% Tween 80 and 10% albumin-dextrose-catalase (ADC) at 37 °C in standing 25 cm^2^ vented tissue culture flasks and subcultured in 75 cm^2^ vented tissue culture flasks. DNA and RNA were extracted from the same cultures (passage 3–4 from original sputum sample) using the phenol-chloroform-isoamyl alcohol method and the trizol method with bead-beating as previously described^42,43^. The samples were sequenced at the Genome Institute of Singapore. Single-molecule real time (SMRT) sequencing from Pacific Biosciences (PacBio) RSII long read technology was used with the parameter of 6 hours per SMRTcell (PacBio RS II SMRT Cells 8Pac). The library preparation involved the use of the template prep kit 1.0, and the binding chemistry involved the use of DNA/Polymerase binding kit P6. The sequencing kit used was the DNA Sequencing Reagent Kit 4.0.

For RNA sequencing, total RNA extracts were run on the Agilent 4200 Tapestation System (Agilent Technologies, Santa Clara, CA, USA) using the RNA Tapestation Assay to determine the RNA integrity values. TruSeq Stranded mRNA sample preparation was used according to the manufacturer’s instructions for next generation library preparation. Briefly, library preparation started with purification of mRNA using poly-T oligo attached magnetic beads, fragmentation of mRNA, 1st and 2nd strand cDNA synthesis, A-tailing and ligation of adapters with multiplex indexes. Samples were enriched with 15 PCR cycles followed by Agencourt AMPure XP magnetic bead (Beckman Coulter, Brea, CA, USA) clean up as per the manufacturer’s instructions. Quality of cDNA libraries was checked with Agilent D1000 Tapestation Assay (Agilent 4200 Tapestation System, Agilent Technologies, Santa Clara, CA, USA). Next generation sequencing was performed using Illumina Hiseq4000 flow cell, with 2 × 151 base pair-end runs. PhiX was used as a control.

### Bioinformatic and association analysis

PacBio long reads were analysed using the pipelines provided by the SMRT Portal software. Briefly, raw sequence data were aligned to the H37Rv (GCA_000195955.2) reference genome and small variants (SNPs and indels) were called over the consensus sequences. Single nucleotide polymorphisms (SNPs) were used to build the maximum-likelihood phylogenetic tree using *RAxML* software^44^. The Modification and Motif Analysis pipeline was used then for the methylation study and motif finding. Detection of base modification was performed with a minimum QV score of 30 and coverage of 20-fold. Six-methyl-adenine (m6A) was determined within motifs with an inter-pulse duration ratio (IPD ratio) between 3 and 10. Statistical enrichment analysis was performed using *DAVID* software^45^. Functional impairment prediction for proteins harbouring non-synonymous mutations was performed using the *Sorting Intolerant from tolerant* (SIFT) algorithm^46^.

Pair-end short reads generated by Illumina HiSeq technology for RNA sequencing were assessed for quality and trimmed using *Trimmomatic* v0.36^47^. High quality reads were mapped to the H37Rv reference genome (GCA_000195955.2) using the *Burrows-Wheeler Alignment* (*BWA*-mem) v0.7.15 tool^48^. *HTSeq*. 0.9.1^49^ was used to quantify the number of reads per transcript. Lowly expressed genes were filtered out by a minimum count per million (CPM) value of 0.6, equivalent to 10 counts. For differential transcription analysis, counts were then normalised using the trimmed mean of M-values normalization (TMM) method^50^. To compare expression levels between ancient and modern strains as well as for the eQTL studies linked with SNPs and methylation, significant differences were obtained through linear regression tests. Adjusted *p* values for multiple testing were calculated through the Bonferroni and Benjamini-Hochberg corrections for statistical significance. The prediction of promoter regions was performed using Neural Network Promoter Prediction (http://www.fruitfly.org/seq_tools/promoter.html). The EGRIN model from the MTB Network Portal^32^ and the regulatory network map from the TB Database^33^ were used for the study of the association between transcriptional regulators and genes differentially expressed. The allele frequencies of variants identified in the eQTL analysis were calculated in an independent set of ancient and modern strains using a large published dataset (n = 6,218), described previously^26^.

## Supplementary information

Supplementary Info

## Data Availability

All pathogen raw sequencing data is available from the ENA short read archive (accession number PRJEB29197).
