# Supplementary material for: An integrated whole genome analysis of Mycobacterium tuberculosis reveals insights into relationship between its genome, transcriptome and methylome
Source: Sci Rep. 2019 Mar 26;9:5204. doi: 10.1038/s41598-019-41692-2 (PMC6435705; doi:10.1038/s41598-019-41692-2)
Supplement: Supplementary file 1 — Supplementary Info [file 41598_2019_41692_MOESM1_ESM.docx]

**An integrated whole genome analysis of *Mycobacterium tuberculosis* reveals insights into relationship between its genome, transcriptome and methylome**

Paula J. Gomez-Gonzalez^1,*^, Nuria Andreu^1,*^, Jody Phelan^1^, Paola Florez de Sessions^2^, Judith R. Glynn^3^, Amelia C. Crampin ^3,4^, Susana Campino^1^, Philip D. Butcher^5^, Martin L. Hibberd^1,2,**^, Taane G. Clark^1,3,**^

^1^ Faculty of Infectious and Tropical Diseases, London School of Hygiene and Tropical Medicine, London, United Kingdom

^2^ Genome Institute Singapore, Singapore

^3^ Faculty of Epidemiology and Population Health, London School of Hygiene and Tropical Medicine, London, United Kingdom

^4^ Malawi Epidemiology and Intervention Research Unit, Lilongwe, Malawi.

^5^ Institute for Infection & Immunity, St George's University of London, UK

* joint authors

** joint authors

Corresponding author:

Professor Taane G. Clark

Pathogen Molecular Biology Department

Faculty of Infectious and Tropical Diseases

London School of Hygiene and Tropical Medicine, London, United Kingdom

**Supplementary Table S1**

**Characteristics of the strains analysed**

| Isolate ID | Year of  collection | Sub-lineage* | Number of SNPs | Number of transcripts** | HIV status | Age | Gender | INH | STR |
| --- | --- | --- | --- | --- | --- | --- | --- | --- | --- |
| RBB389 | 2008 | 1.1.2 (EAI3; EAI5) | 2751 | 3836 | + | 35 | F | S | S |
| RBB395 | 2009 | 1.1.2 (EAI3; EAI5) | 2612 | 3950 | + | 37 | F | S | S |
| RBB398 | 2009 | 1.1.2 (EAI3; EAI5) | 2583 | 3961 | + | 31 | M | S | S |
| RBB383 | 2007 | 1.1.3 (EAI6) | 2320 | 3934 | + | 35 | M | S | S |
| RBB385 | 2007 | 1.1.3 (EAI6) | 2619 | 3948 | + | 40 | M | S | S |
| RBB388 | 2008 | 1.2.2 (EAI1) | 2632 | 3935 | - | 36 | F | S | S |
| RBB394 | 2009 | 1.2.2 (EAI1) | 2612 | 3765 | + | 53 | M | R | S |
| RBB397 | 2009 | 1.2.2 (EAI1) | 2643 | 3953 | + | 33 | F | S*** | S*** |
| RBB401 | 2010 | 2.2.1 (Beijing) | 1651 | 3891 | + | 46 | F | S | S |
| RBB402 | 2010 | 2.2.1 (Beijing) | 1676 | 3937 | - | 74 | M | S | S |
| RBB384 | 2007 | 4.1.1.3 (Haarlem X1, X3) | 1473 | 3931 | + | 43 | M | S | S |
| RBB399 | 2010 | 4.1.1.3 (Haarlem X1, X3) | 1423 | 3934 | + | 26 | F | S | S |
| RBB404 | 2004 | 4.1.1.3 (Haarlem X1, X3) | 1217 | 3950 | - | 33 | F | S | S |
| RBB387 | 2007 | 4.1.2 (X-type) | 1482 | 3933 | + | 45 | M | S | S |
| RBB392 | 2008 | 4.1.2 (X-type) | 1426 | 3953 | + | 63 | M | S | S |
| RBB386 | 2007 | 4.3.3 (LAM) | 1298 | 3345 | + | 50 | F | S | S |
| RBB396 | 2009 | 4.3.3 (LAM) | 1102 | 3950 | - | 33 | M | S | S |
| RBB403 | 2003 | 4.3.4.2.1 (LAM) | 1063 | 3929 | - | 67 | M | S | S |
| RBB391 | 2008 | 4.8 (T) | 857 | 3950 | + | 34 | M | S | S |
| RBB390 | 2008 | 4.9 (T1-H37Rv) | 528 | 3967 | + | 35 | M | R | R |
| RBB393 | 2008 | 4.9 (T1-H37Rv) | 635 | 3083 | - | 49 | F | S | S |
| RBB400 | 2010 | 4.9 (T1-H37Rv) | 634 | 3965 | + | 18 | M | S*** | S*** |

* Lineages are underlined; isoniazid (INH) and streptomycin (STR) drug susceptibility test (R: resistant; S: susceptible); ** number of genes transcribed with at least 10 counts; *** inferred by whole-genome sequencing.

**Supplementary Table S2**

**105 genes found to be differentially expressed between ancient (lineage 1) and modern (lineages 2 and 4) isolates**

| Gene | Log2 Fold-change  (ancient vs modern) | Adjusted *p* value |
| --- | --- | --- |
| *Rv0028* | 0.930 | 3.48x10^-4^ |
| *Rv0060* | 1.260 | 1.22x10^-5^ |
| *ephF* (*Rv0134*) | -1.215 | 1.53x10^-3^ |
| *Rv0192* | 0.240 | 5.67x10^-3^ |
| *Rv0193c* | 1.663 | 2.94x10^-3^ |
| *nirB* (*Rv0252*) | 2.701 | 7.38x10^-4^ |
| *nirD* (*Rv0253*) | 2.661 | 5.12x10^-3^ |
| *Rv0273c* | -1.642 | 8.89x10^-8^ |
| *Rv0275c* | -2.572 | 9.48x10^-5^ |
| *Rv0276* | -5.341 | 1.89x10^-9^ |
| *PPE3* (*Rv0280*) | -2.303 | 5.11x10^-3^ |
| *iniB* (*Rv0341*) | -4.054 | 6.23x10^-5^ |
| *iniA* (*Rv0342*) | -2.367 | 3.36x10^-4^ |
| *iniC* (*Rv0343*) | -2.388 | 3.35x10^-7^ |
| *icl1* (*Rv0467*) | 2.587 | 2.85x10^-2^ |
| *fadB2* (*Rv0468*) | 1.426 | 6.48x10^-3^ |
| *umaA* (*Rv0469*) | 1.563 | 9.27x10^-7^ |
| *Rv0520* | -2.961 | 3.65x10^-6^ |
| *galTb* (*Rv0619*) | 3.691 | 3.84x10^-5^ |
| *galK* (*Rv0620*) | 5.493 | 2.81x10^-6^ |
| *recB* (*Rv0630c*) | 1.550 | 4.31x10^-5^ |
| *Rv0687* | -1.190 | 4.83x10^-5^ |
| *rplN* (*Rv0714*) | 0.509 | 1.17x10^-2^ |
| *Rv0826* | -3.974 | 3.82x10^-2^ |
| *rpfA* (*Rv0867c*) | 1.647 | 3.96x10^-4^ |
| *Rv0906* | -1.416 | 7.57x10^-5^ |
| *Rv0966c* | -1.646 | 1.14x10^-3^ |
| *Rv0997* | 0.981 | 5.84x10^-3^ |
| *Rv1101c* | -1.319 | 2.58x10^-3^ |
| *narH* (*Rv1162*) | -2.399 | 6.90x10^-3^ |
| *narJ* (*Rv1163*) | -2.577 | 2.19x10^-3^ |
| *narI* (*Rv1164*) | -1.682 | 1.78x10^-4^ |
| *deaD* (*Rv1253*) | -1.116 | 1.41x10^-3^ |
| *Rv1261c* | 0.313 | 4.52x10^-2^ |
| *pknH* (*Rv1266c*) | 1.069 | 5.04x10^-5^ |
| *Rv1319c* | -0.796 | 1.58x10^-2^ |
| *PE_PGRS25* (*Rv1396c*) | -2.440 | 2.14x10^-2^ |
| *vapC10* (*Rv1397c*) | -3.070 | 6.82x10^-5^ |
| *vapB10* (*Rv1398c*) | -3.289 | 1.86x10^-4^ |
| *Rv1503c* | 1.836 | 1.98x10^-3^ |
| *Rv1504c* | 2.443 | 5.41x10^-3^ |
| *Rv1505c* | 2.284 | 8.78x10^-3^ |
| *Rv1524** | -2.051 | 2.57x10^-7^ |
| *wbbL2** (*Rv1525*) | -7.438 | 1.67x10^-9^ |
| *plsB1* (*Rv1551*) | 2.205 | 1.391x10^-4^ |
| *cya* (*Rv1625c*) | -0.956 | 3.67x10^-2^ |
| *Rv1627c* | -0.585 | 4.60x10^-2^ |
| *malQ* (*Rv1781c*) | 1.548 | 2.24x10^-2^ |
| *PE_PGRS34* (*Rv1840c*) | -1.116 | 5.21x10^-3^ |
| *Rv1842c* | -0.860 | 9.05x10^-3^ |
| *Rv1895* | 2.647 | 3.33x10^-6^ |
| *lppD (Rv1899c)* | -4.085 | 1.19x10^-15^ |
| *PPE35* (*Rv1918c*) | -1.220 | 4.30x10^-3^ |
| *fadD31* (*Rv1925*) | -2.890 | 7.10x10^-13^ |
| *Rv1976c* | 0.753 | 1.08x10^-2^ |
| *Rv2059* | -1.244 | 1.14x10^-2^ |
| *Rv2159c* | -5.676 | 3.92x10^-3^ |
| *Rv2160A* | -5.798 | 7.13x10^-4^ |
| *Rv2161c* | -5.754 | 5.41x10^-7^ |
| *PE_PGRS38* (*Rv2162c*) | -4.112 | 9.36x10^-12^ |
| *pimB* (*Rv2188c*) | -3.360 | 7.74x10^-6^ |
| *Rv2271* | 0.950 | 6.28x10^-4^ |
| *Rv2272* | 0.984 | 3.94x10^-5^ |
| *Rv2282c* | 0.801 | 1.96x10^-2^ |
| *narK1* (*Rv2329c*) | 1.844 | 3.99x10^-2^ |
| *Rv2337c* | -2.377 | 6.43x10^-4^ |
| *PE_PGRS42* (*Rv2487c*) | -1.238 | 3.06x10^-2^ |
| *Rv2652c** | -2.103 | 4.43x10^-2^ |
| *Rv2653c** | -2.666 | 4.22x10^-2^ |
| *Rv2658c** | -2.663 | 4.22x10^-2^ |
| *Rv2712c* | 1.896 | 1.98x10^-9^ |
| *Rv2719c* | 1.951 | 1.99x10^-9^ |
| *Rv2765* | 4.360 | 1.14x10^-8^ |
| *vapB22* (*Rv2830c*) | 1.551 | 2.03x10^-3^ |
| *amt* (*Rv2920c*) | 1.091 | 3.90x10^-2^ |
| *Rv2994* | -1.690 | 3.48x10^-7^ |
| *Rv3007c* | -2.168 | 2.43x10^-9^ |
| *virS* (*Rv3082c*) | 3.052 | 3.34x10^-3^ |
| *PPE51* (*Rv3136*) | 2.571 | 1.23x10^-6^ |
| *Rv3137* | 2.665 | 2.45x10^-4^ |
| *pflA* (*Rv3138*) | 1.119 | 2.17x10^-2^ |
| *PPE52* (*Rv3144c*) | -0.995 | 1.33x10^-2^ |
| *Rv3165c* | 0.592 | 6.34x10^-3^ |
| *Rv3167c* | 2.247 | 2.70x10^-3^ |
| *Rv3168* | 2.106 | 4.94x10^-2^ |
| *Rv3169* | 2.295 | 2.65x10^-4^ |
| *Rv3233c* | -1.733 | 6.25x10^-7^ |
| *Rv3446c* | 2.177 | 4.47x10^-3^ |
| *mce4A* (*Rv3499c*) | 0.616 | 1.96x10^-3^ |
| *yrbE4B* (*Rv3500c*) | 1.697 | 5.17x10^-4^ |
| *PE_PGRS57** (*Rv3514*) | -2.748 | 1.52x10^-6^ |
| *Rv3527* | -1.003 | 4.06x10^-2^ |
| *PE33* (*Rv3650*) | 1.136 | 3.74x10^-2^ |
| *PE_PGRS60* (*Rv3652*) | 3.675 | 3.69x10^-11^ |
| *PE_PGRS61* (*Rv3653*) | 4.647 | 1.49x10^-9^ |
| *Rv3679* | -4.162 | 3.05x10^-12^ |
| *Rv3680* | -3.146 | 7.88x10^-11^ |
| *Rv3695* | -2.202 | 1.58x10^-9^ |
| *Rv3740c* | 1.246 | 2.69x10^-4^ |
| *Rv3741c* | 1.682 | 2.60x10^-2^ |
| *Rv3742c* | 2.550 | 3.70x10^-3^ |
| *accD4* (*Rv3799c*) | -1.195 | 7.26x10^-3^ |
| *pks13* (*Rv3800c*) | -1.390 | 6.95x10^-4^ |
| *PE_PGRS62* (*Rv3812*) | 1.249 | 1.82x10^-7^ |
| *Rv3915* | 0.244 | 5.50x10^-3^ |

***** Genes deleted in ancient (lineage 1) isolates.

Adjusted *p* value obtained by Bonferroni correction.

**Supplementary Table S3**

**42 genes found to be under-expressed (adjusted *p* < 0.05) and associated with large genomic deletions**

| Gene | Lineage/sub-lineage with the deletion |
| --- | --- |
| *Rv0072* | L2 |
| *Rv0073* | L2 |
| *msrA* (*Rv0137c*) | 4.3.4.2.1 |
| *Rv0195* | 4.1.2 |
| *aac* (*Rv0262c*) | 1.2.2* |
| *Rv0265c* | 1.2.2* |
| *oplA* (*Rv0266c*) | 1.2.2* |
| *Rv1524* | 1 |
| *wbbL2* (*Rv1525*) | 1 |
| *gabD2* (*Rv1731*) | 1.1.3 |
| *PE18* (*Rv1788*) | 1.2.2* |
| *PE26* (*Rv1789*) | 1.2.2* |
| *Rv1993c* | 4.3.4.2.1 |
| *cmtR* (*Rv1994c*) | 4.3.4.2.1 |
| *plcC* (*Rv2349c*) | 1.1.3 |
| *plcB* (*Rv2350c*) | 1.1.3 |
| *plcA* (*Rv2351c*) | 1.1.3 |
| *PPE39* (*Rv2353c*) | 2 |
| *Rv2645* | 1* |
| *Rv2646* | 1* |
| *Rv2647* | 1* |
| *Rv2651c* | 1* |
| *Rv2652c* | 1* |
| *Rv2655c* | 1* |
| *Rv2656c* | 1* |
| *Rv2657c* | 1* |
| *Rv2658c* | 1* |
| *Rv2819c* | 2 |
| *PPE55* (*Rv3347c*) | 4.3.4.2.1 |
| *Rv3349c* | 4.3.4.2.1 |
| *PPE56* (*Rv3350c*) | 4.3.4.2.1 |
| *Rv3351c* | 4.3.4.2.1 |
| *lytB1* (*Rv3382c*) | 2* |
| *cmaA1* (*Rv3392c*) | 4.9* |
| *PPE58* (*Rv3426*) | 1,2,4.8,4.9 |
| *Rv3468c* | 4.8 |
| *mhpE* (*Rv3469c*) | 4.8 |
| *ilvB2* (*Rv3470c*) | 4.8 |
| *Rv3471c* | 4.8 |
| *Rv3472* | 4.8 |
| *bpoA* (*Rv3473c*) | 4.8 |
| *kgtP* (*Rv3476c*) | 4.8 |

* Not all the clinical isolates from the lineage or sub-lineage.

**Supplementary Table S4**

**76 genes found to be differentially expressed (adjusted *p* < 0.05) through eQTL analysis**

| Gene | Number of SNPs associated** | Lineage/sub-lineage | Regulation |
| --- | --- | --- | --- |
| *Rv0273c* | 798 | 1 | Down |
| *Rv0276* | 790 | 1 | Down |
| *iniC* (*Rv0343*) | 790 | 1 | Down |
| *umaA* (*Rv0469*) | 790 | 1 | Up |
| *Rv0520* | 790 | 1 | Down |
| *Rv0576* | 4 | 1.1.2*, 1.2.2* | Up |
| *mce2R* (*Rv0586*) | 1 | 1.1.2*, 1.1.3* | Up |
| *mce2D* (*Rv0592*) | 169 | 1, 2 | Up |
| *galK* (*Rv0620*) | 790 | 1 | up |
| *recB* (*Rv0630c*) | 7 | 1* | Up |
| *mazF2* (*Rv0659c*) | 297 | 4.1.2 | Down |
| *mazE2* (*Rv0660c*) | 297 | 4.1.2 | Down |
| *Rv0687* | 84 | 1 | Down |
| *Rv0750* | 368 | 4.1.1.3 | Up |
| *Rv0958* | 398 | 1.1.3 | Up |
| *Rv0959* | 398 | 1.1.3 | Up |
| *Rv1096* | 93 | 1, 2, 4.1, 4.3, 4.8 | Up |
| *Rv1101c* | 169 | 1, 2 | Down |
| *bpoB* (*Rv1123c*) | 368 | 4.1.1.3 | Down |
| *narH* (*Rv1162*) | 6 | 1* | Down |
| *narJ* (*Rv1163*) | 7 | 1* | Down |
| *narI* (*Rv1164*) | 7 | 1* | Down |
| *Rv1318c* | 137 | 4.3 | Up |
| *Rv1371* | 93 | 1, 2, 4.1, 4.3, 4.8 | Up |
| *vapC10* (*Rv1397c*) | 7 | 1* | Down |
| *vapB10* (*Rv1398c*) | 7 | 1* | Down |
| *Rv1429* | 368 | 4.1.1.3 | Up |
| *bisC* (*Rv1442*) | 484 | 1.2.2* | Up |
| *Rv1489* | 297 | 1.2.2* | Down |
| *Rv1489A* | 297 | 1.2.2* | Down |
| *Rv1490* | 297 | 1.2.2* | Down |
| *Rv1491c* | 297 | 1.2.2* | Down |
| *Rv1764* | 94 | 1, 2, 4.1, 4.3, 4.8 | Down |
| *Rv1895* | 798 | 1 | Up |
| *lppD* (*Rv1899c*) | 791 | 1 | Down |
| *fadD31* (*Rv1925*) | 805 | 1 | Down |
| *Rv1976c* | 169 | 1, 2 | Up |
| *vapC36* (*Rv1982c*) | 121 | 4.1 | Up |
| *Rv2077c* | 127 | 1, 2, 4.1, 4.3 | Down |
| *Rv2159c* | 170 | 1, 2 | Down |
| *Rv2160A* | 169 | 1, 2 | Down |
| *Rv2161c* | 963 | 1, 2 | Down |
| *PE_PGRS38* (*Rv2162c*) | 790 | 1 | Down |
| *Rv2271* | 1 | 1 | Up |
| *Rv2324* | 4 | 1.1.2*, 1.2.2* | Up |
| *Rv2337c* | 7 | 1* | Down |
| *vapB38* (*Rv2493*) | 121 | 4.1 | Up |
| *vapC38* (*Rv2494*) | 121 | 4.1 | Up |
| *arsC* (*Rv2643*) | 226 | 1.1.3* | Up |
| *Rv2712c* | 790 | 1 | Up |
| *Rv2719c* | 790 | 1 | Up |
| *Rv2765* | 797 | 1 | Up |
| *Rv2915c* | 226 | 1.1.3* | Up |
| *Rv2972c* | 1 | 1, 2 | Up |
| *recG* (*Rv2973c*) | 1 | 1, 2 | Up |
| *Rv2974c* | 169 | 1, 2 | Up |
| *Rv2994* | 790 | 1 | Down |
| *Rv3007c* | 790 | 1 | Down |
| *PPE51* (*Rv3136*) | 797 | 1* | Up |
| *Rv3169* | 7 | 1* | Up |
| *Rv3233c* | 790 | 1 | Down |
| *Rv3322c* | 1 | 4.1.2, 4.9 | Down |
| *moaC3* (*Rv3324c*) | 93 | 4.9 | Down |
| *spoU* (*Rv3366*) | 584 | 2 | Up |
| *fadD17* (*Rv3506*) | 177 | 4.9* | Up |
| *PE_PGRS60* (*Rv3652*) | 790 | 1 | Up |
| *PE_PGRS61* (*Rv3653*) | 790 | 1 | Up |
| *Rv3679* | 791 | 1 | Down |
| *Rv3680* | 790 | 1 | Down |
| *Rv3695* | 790 | 1 | Down |
| *Rv3706c* | 198 | 1.1.3* | Up |
| *Rv3750c* | 127 | 4.8, 4.9 | Up |
| *tcrX* (*Rv3765c*) | 198 | 1.1.3* | Up |
| *PE_PGRS62* (*Rv3812*) | 790 | 1 | Up |
| *Rv3829c* | 584 | 2 | Up |
| *Rv3830c* | 584 | 2 | up |

* Not all the clinical isolates from the lineage or sub-lineage.

** Number of common SNPs in isolates with a gene over- or under-expressed compared to the rest of isolates not carrying the SNPs. All the lineage or sub-lineage specific SNPs are therefore associated with genes differentially expressed by lineage or sub-lineage.

**Supplementary Table S5**

**Functional SNPs located in the upstream intergenic region, upstream gene or within the gene associated with differential expression (*cis*-eQTLs, adjusted *p* < 0.05)**

|  | Transcript differentially expressed | Annotation | SNP | Position SNP | | | Regulation | Strain  Lineage | Allele frequency** | |
| --- | --- | --- | --- | --- | --- | --- | --- | --- | --- | --- |
|  |  |  |  | **Gene** | **Distance (bp) from start codon** | **Promoter (P)/TSS** |  |  | **Ancient** | **Modern** |
| SNPs in upstream Intergenic region (IGR) | *Rv0068* | 3 | C75231T | IGR | -70 | P | Up | 4.1.2 | 0 | 0.009 |
|  | *Rv0193c* | 1 | G226676A | IGR | -105 | - | Up | 1 | 0.973 | 0 |
|  | *gpdA1* | 4 | T655986G | IGR | -37 | P | Up | 1,2 | 0.976 | 0.324 |
|  | *Rv0669c* | 3 | T769663G | IGR | -66 | P | Down | 4.3.3 | 0 | 0.050 |
|  | *Rv0750* | 1 | C841924T | IGR | -109 | - | Up | 4.1.1.3 | 0.003 | 0.038 |
|  | *Rv0958* | 3 | C1069871T | IGR | -12 | P | Up | 1.1.3 | 0.220 | 0 |
|  | *Rv1096* | 3 | T1224367C | IGR | -18 | P | Down | 1,2,4.1,4.3,4.8 | 1 | 0.976 |
|  | *Rv1503c* | 1 | A1694547C | IGR | -3 | - | Up | 1 | 0.973 | 0 |
|  | *fadD31* | 4 | T2177073C | IGR | -14 | TSS/P | Down | 1 | 0.973 | 0 |
|  | *PE_PGRS38* | 7 | A2424864G | IGR | -18 | TSS | Down | 1 | 0.973 | 0 |
|  | *Rv2712c* | 1 | C3025431T | IGR | -103 | P | Up | 1 | 0.971 | 0 |
|  | *vapB22* | 5 | T3137237C | IGR | -13 | P | Up | 1 | 0.973 | 0 |
|  | *Rv2923c* | 1 | G3238516A | IGR | -17 | - | Up | 4.1.2 | 0 | 0.009 |
|  | *Fpg* | 8 | G3239476A | IGR | -6 | - | Up | 4.1.2 | 0 | 0.008 |
|  | *Rv3695* | 2 | T4137190C | IGR | -16 | - | Down | 1 | 0.973 | 0 |
| SNPs within gene or upstream gene | *Rv0060* | 1 | C64028T | *Rv0060* | 119 | - | Up | 1 | 0.973 | 0.002 |
|  | *ephF* | 5 | G162226A | *ephF* | 455 | - | Down | 1 | 0973 | 0 |
|  | *Rv0193c* | 1 | C225668T | *Rv0193c* | 903 | - | Up | 1 | 0.971 | 0 |
|  | *Rv0275c* | 6 | G331588A | *Rv0275c* | 70 | - | Down | 1 | 0.973 | 0 |
|  | *Rv0276* | 1 | G331588A | *Rv0275c* | 160 | - | Down | 1 | 0.973 | 0 |
|  | *PPE3* | 7 | C339508T | *PPE3* | 144 | - | Down | 1 | 0.968 | 0 |
|  | *PPE5* | 7 | C370229T | *PPE5* | 2535 | - | Down | 1.1.3 | 0.230 | 0 |
|  | *Rv0326* | - | T392261C | *Rv0325* | -12 | - | Up | 1,2 | 0.978 | 0.324 |
|  | *iniA* | 2 | T412280G | *iniA* | 1442 | - | Down | 1 | 0.973 | 0 |
|  | *Rv0376c* | 1 | T454295C | *Rv0376c* | 77 | - | Up | 1,2,4.1,4.3.4,4.8,4.9 | 1 | 0.994 |
|  | *Rv0377* | 6 | T454295C | *Rv0376c* | -126 | - | Up | 1,2,4.1,4.3.4,4.8,4.9 | 1 | 0.994 |
|  | *umaA* | 4 | C560664T | *umaA* | 776 | - | Up | 1 | 0.973 | 0 |
|  |  |  | A560666G |  | 778 | - |  |  | 0.973 | 0 |
|  | *mce2R* | 6 | C684611T | *mce2R* | 201 | - | Up | 1.1.2* | 0.024 | 0 |
|  | *mce2C* | 5 | A690450C | *mce2C* | 1391 | - | Up | 1,2 | 0.976 | 0.324 |
|  | *mce2D* | 6 | A690450C | *mce2C* | -51 | - | Up | 1,2 | 0.976 | 0.324 |
|  | *recB* | 8 | G722852A | *recB* | 2161 | - | Up | 1 | 0.973 | 0 |
|  | *Rv0669c* | 3 | A768395G | *Rv0669c* | 1202 | - | Down | 4.3.3* | 0 | 0 |
|  | *rplN* | 8 | C811492G | *rplN* | 119 | - | Up | 1 | 0.970 | 0 |
|  | *Rv0750* | 1 | C842111G | *Rv0750* | 78 | - | Up | 4.1.1.3 | 0.029 | 0.060 |
|  | *Rv0906* | 1 | C1009490T | *Rv0906* | 546 | - | Down | 1 | 0.971 | 0 |
|  | *Rv0966c* | 1 | C1077754T | *Rv0966c* | 81 | - | Down | 1 | 0.971 | 0 |
|  | *Rv1048c* | 1 | G1171183A | *Rv1048c* | 970 | - | Up | 1.2.2* | 0.021 | 0 |
|  | *bpoB* | 5 | G1246845A | *bpoB* | 207 | - | Down | 4.1.13 | 0 | 0.003 |
|  | *deaD* | 8 | A1400396G | *deaD* | 426 | - | Down | 1 | 0.971 | 0 |
|  | *Rv1318c* | 3 | G1480024T | *Rv1318c* | 800 | - | Up | 4.3 | 0.013 | 0.277 |
|  | *Rv1319c* | 3 | T1481602G | *Rv1319c* | 899 | - | Down | 1 | 0.970 | 0 |
|  | *vapC10* | 5 | T1574206C | *vapC10* | 307 | - | Down | 1 | 0.970 | 0 |
|  | *Rv1429* | 1 | C1605149T | *Rv1429* | 271 | - | Up | 4.1.1.3 | 0.005 | 0.049 |
|  | *bisC* | 3 | G1619841A | *bisC* | 50 | - | Up | 1.2.2* | 0.157 | 0 |
|  | *Rv1505c* | 1 | G1695674A | *Rv1505c* | 272 | - | Up | 1 | 0.973 | 0 |
|  | *vapB11* | 5 | G1764812A | *vapB11* | 57 | - | Up | 4.3.3* | 0 | 0 |
|  | *vapC11* | 5 | G1764812A | *vapB11* | -167 | - | Up | 4.3.3* | 0 | 0 |
|  | *Rv1773c* | 6 | G2007502A | *Rv1773c* | 264 | - | Up | 4.1 | 0.003 | 0.176 |
|  | *Rv1776c* | 6 | G2010096T | *Rv1776c* | 459 | - | Up | 1.2.2* | 0.019 | 0 |
|  | *lldD2* | 3 | C2123181T | *Rv1873* | -30 | - | Up | 4.1.2 | 0.022 | 0.025 |
|  | *lppD* | 2 | A2145878G | *lppD* | 367 | - | Down | 1* | 0.973 | 0 |
|  | *fadD31* | 4 | G2177968T | *fadD31* | 881 | - | Down | 1 | 0.973 | 0 |
|  | *Rv1982c* | 5 | A2225456T | *Rv1982c* | 376 | - | Up | 4.1 | 0.003 | 0.176 |
|  | *Rv2036* | 3 | C2282058T | *Rv2035* | -41 | - | Up | 1.2.2* | 0.157 | 0 |
|  | *Rv2077c* | 2 | A2334007G | *Rv2077c* | 287 | - | Down | 1,2,4.1,4.3 | 1 | 0.882 |
|  | *Rv2159c* | 1 | A2421816G | *Rv2160A* | -151 | - | Down | 1,2 | 0.977 | 0.323 |
|  | *Rv2160A* | 6 | A2421816G | *Rv2160A* | 462 | - | Down | 1,2 | 0.977 | 0.323 |
|  | *PE_PGRS38* | 7 | C2423785T | *PE_PGRS38* | 1053 | - | Down | 1 | 0.962 | 0.001 |
|  | *pimB* | 4 | G2450045A | *pimB* | 1105 | - | Down | 1 | 0.971 | 0 |
|  |  |  | C2451081G |  | 69 | - |  |  | 0.973 | 0 |
|  | *Rv2263* | 3 | C2536599T | *Rv2263* | 958 | - | Down | 2 | 0.003 | 0.126 |
|  | *plcC* | 3 | G2627377T | *plcC* | 1321 | - | Down | 1.1.3 | 0.232 | 0 |
|  | *Rv2719c* | 2 | A3031285T | *Rv2719c* | 252 | - | Up | 1 | 0.973 | 0 |
|  | *Rv2765* | 3 | C3074830T | *Rv2765* | 194 | - | Up | 1 | 0.973 | 0 |
|  | *Rv2994* | 2 | G3351472A | *Rv2994* | 203 | - | Down | 1 | 0.973 | 0 |
|  | *Rv3027c* | 1 | G3386782A | *Rv3027c* | 137 | - | Up | 4.1.2 | 0 | 0.009 |
|  | *Rv3081* | 1 | C3446699G | *Rv3081* | 659 | - | Up | 2 | 0.011 | 0.159 |
|  | *virS* | 5 | A3447480C | *virS* | 946 | TSS | Up | 1 | 0.973 | 0 |
|  | *Rv3167c* | 6 | G3536008T | *Rv3167c* | 49 | - | Up | 1 | 0.973 | 0 |
|  | *Rv3180c* | 1 | C3549576A | *Rv3180c* | 112 | - | Up | 1.1.2* | 0.013 | 0 |
|  | *lhr* | 8 | C3678298T | *lhr* | 1523 | - | Up | 4.1.2* | 0 | 0.004 |
|  | *PPE55* | 7 | A3746409G | *PPE55* | 6775 | - | Up | 1,2,4.1,4.3.3,4.8,4.9 | - | - |
|  |  |  | A3752207G |  | 977 | - |  |  | - | - |
|  | *spoU* | 8 | GG3778011AT | *spoU* | 274 | - | Up | 2 | 0.003 | 0.147 |
|  | *PPE57* | 7 | T3842425A | *PPE57* | 186 | - | Down | 4.1/4.3 | - | - |
|  |  |  | AG3842581GT |  | 342 | - |  |  | - | - |
|  | *Rv3446c* | 1 | G3864041A | *Rv3446c* | 490 | - | Up | 1 | 0.970 | 0 |
|  | *kgtP* | 2 | A3892671G | *kgtP* | 1049 | - | Up | 1,2,4.1,4.3,4.9 | 1 | 0.999 |
|  | *yrbE4B* | 5 | G3920109T | *yrbE4A* | -47 | - | Up | 1 | 0.971 | 0 |
|  | *fadD17* | 4 | C3925702T | *fadD17* | 812 | - | Up | 4.9* | 0.003 | 0 |
|  | *PPE65* | 7 | A4060742G | *PPE65* | 1147 | - | Down | 4.3.3* | 0 | 0 |
|  | *PE_PGRS60* | 7 | G4093719A | *PE_PGRS60* | 87 | - | Up | 1 | 0.971 | 0 |
|  | *Rv3679* | 2 | T4119246C | *Rv3679* | 470 | - | Down | 1 | 0.968 | 0 |
|  | *PE_PGRS62* | 7 | G4277032C | *PE_PGRS62* | 461 | - | Up | 1 | 0.971 | 0 |

* Only one or two samples from the lineage out of the 3 analysed; ** Allele frequency refers to the proportion of strains harbouring the SNP in a larger data set (n = 6,218)^50^.

**Supplementary Table S6**

**Genes differentially expressed (adjusted *p* < 0.05) associated with transcriptional regulators carrying candidate impairing mutations**

| Transcriptional Regulator | Mutation | Genes Differentially Expressed | Regulation | Lineage | Allele frequency** | | |
| --- | --- | --- | --- | --- | --- | --- | --- |
|  |  |  |  |  | **Ancient** | **Modern** | |
| *Rv0275c* | S24L | *Rv0276, Rv0520, Rv2162c, Rv0275c*, Rv0826* | Down | 1 | 0.973 | | 0 |
| *ramB* | Q121R  P91Q | *Rv1895, Rv3233c, Rv1164*, Rv1163*, Rv1162** | Up/Down | 1 | 0.973 | | 0 |
| *Rv1776c* | R154S | *Rv1048c, Rv1776c, Rv3136* | Up | 1.2.2 | 0.019 | | 0 |
| *Rv3167c* | P17Q | *Rv1895* | Up | 1 | 0.973 | | 0 |
| *Rv3249c* | T154A | *Rv1429, Rv1123c* | Up/Down | 4.1.1.3 | 0.003 | | 0.049 |

* Genes that are differentially expressed but didn’t reach the cut off (adjusted *p* < 0.05).

** Allele frequency refers to the proportion of strains harbouring the mutation in a larger data set (n = 6,218)^50^.

**Supplementary Table S7**

**Mutations found in anti-sigma factors (as per H37Rv reference annotation) related with differential gene expression**

| Sigma Factor | Mutation | Lineage | Genes differentially expressed | Regulation | Allele frequency | | |
| --- | --- | --- | --- | --- | --- | --- | --- |
|  |  |  |  |  | **Ancient** | **Modern** | |
| *rseA* | A23T | 4.1.1.3 | *Rv0750, Rv1429* | Up | 0.003 | | 0.049 |
| *rskA* | E81D | 2 | *spoU, Rv3829c, Rv3830c* | Up | 0.003 | | 0.126 |
| *rsfA* | L125R | 1.2.2* | *bisC* | Up | 0.148 | | 0 |

* Not all the clinical isolates from the lineage or sub-lineage; Allele frequency refers to the proportion of strains harbouring the mutation in a larger data set (n = 6,218)^50^.

**Supplementary Table S8**

**Fractions of methylation for each identified motif**

| **Sample** | **CTCCAG** | **CTGGAG** | **GATN_4_RTAC** | **GTAYN_4_ATC** | **CACGCAG** | **Lineage** |
| --- | --- | --- | --- | --- | --- | --- |
| RBB389 | 1795/1927 (0.93) | 1704/1930 (0.88) | 311/347 (0.9) | 307/348 (0.88) | 749/804 (0.93) | 1.1.2 |
| RBB398 | 1749/1934 (0.9) | 1632/1937 (0.84) | 304/356 (0.85) | 289/355 (0.81) | 739/811 (0.91) | 1.1.2 |
| RBB395 | 6/1929 (0.003) | 0/1930 (0) | 296/351 (0.84) | 289/348 (0.83) | 759/806 (0.94) | 1.1.2 |
| RBB383 | 211/1912 (0.11) | 193/1911 (0.1) | 36/352 (0.1) | 31/351 (0.08) | 1/803 (0.001) | 1.1.3 |
| RBB385 | 1580/1941 (0.81) | 1394/1940 (0.72) | 272/361 (0.75) | 269/362 (0.74) | 9/817 (0.01) | 1.1.3 |
| RBB388 | 1366/1924 (0.71) | 1246/1925 (0.65) | 249/347 (0.72) | 237/348 (0.68) | 640/804 (0.8) | 1.2.2 |
| RBB394 | 1208/1929 (0.63) | 1134/1929 (0.59) | 255/355 (0.72) | 246/353 (0.69) | 0/808 (0) | 1.2.2 |
| RBB397 | 1618/1927 (0.84) | 1475/1928 (0.77) | 294/347 (0.85) | 280/347 (0.81) | 720/805 (0.89) | 1.2.2 |
| RBB401 | 0/1937 (0) | 0/1935 (0) | 201/360 (0.56) | 178/360 (0.5) | 485/815 (0.6) | 2.2.1 |
| RBB402 | 2/1938 (0.001) | 4/1938 (0.002) | 304/360 (0.85) | 302/360 (0.84) | 690/815 (0.84) | 2.2.1 |
| RBB384 | 1791/1930 (0.93) | 1709/1934 (0.88) | 307/355 (0.86) | 305/357 (0.85) | 766/810 (0.95) | 4.1.1.3 |
| RBB399 | 1772/1933 (0.92) | 1685/1934 (0.87) | 305/359 (0.85) | 303/359 (0.84) | 756/810 (0.93) | 4.1.1.3 |
| RBB404 | 1524/1930 (0.79) | 1400/1930 (0.73) | 273/355 (0.77) | 252/358 (0.7) | 701/810 (0.87) | 4.1.1.3 |
| RBB387 | 1767/1942 (0.91) | 1622/1943 (0.83) | 306/361 (0.85) | 284/361 (0.79) | 761/819 (0.93) | 4.1.2 |
| RBB392 | 1774/1942 (0.91) | 1712/1943 (0.88) | 305/361 (0.84) | 302/360 (0.84) | 756/819 (0.92) | 4.1.2 |
| RBB386 | 104/1930 (0.05) | 109/1933 (0.06) | 0/361 (0) | 0/362 (0) | 672/812 (0/83) | 4.3.3 |
| RBB396 | 1497/1944 (0.77) | 1332/1943 (0.69) | 0/363 (0) | 0/363 (0) | 732/819 (0.89) | 4.3.3 |
| RBB403 | 1586/1927 (0.82) | 1408/1928 (0.73) | 0/362 (0) | 0/362 (0) | 659/816 (0.89) | 4.3.3 |
| RBB391 | 1754/1935 (0.91) | 1643/1936 (0.85) | 0/357 (0) | 0/358 (0) | 0/813 (0) | 4.8 |
| RBB390 | 1827/1946 (0.94) | 1760/1947 (0.9) | 0/363 (0) | 0/363 (0) | 0/819 (0) | 4.9 |
| RBB393 | 1718/1943 (0.88) | 1593/1945 (0.82) | 0/360 (0) | 0/361 (0) | 0/819 (0) | 4.9 |
| RBB400 | 1812/1946 (0.93) | 1751/1946 (0.9) | 0/362 (0) | 0/362 (0) | 0/820 (0) | 4.9 |

Methylated motifs/Total motifs (fraction of methylation). Cells coloured in red correspond to isolates with non-methylated motifs. Underlined in the motif shows the methylated nucleotide (m6A).

**Supplementary Table S9**

**Mutations found in each *Mtb* MTase**

| **Sample** | ***mamA*** | ***hsdM*** | ***mamB*** | **Lineage** |
| --- | --- | --- | --- | --- |
| RBB389 | - | V93V | W47R, D154G, 1515delC | 1.1.2 |
| RBB398 | - | V93V | W47R, D154G | 1.1.2 |
| RBB395 | **Q340K, 121delG** | V93V | W47R, D154G, 1515delC | 1.1.2 |
| RBB383 | - | V93V | W47R, D154G, **S253L** | 1.1.3 |
| RBB385 | - | V93V | W47R, D154G, **S253L** | 1.1.3 |
| RBB388 | - | V93V, T450T, K211Q | W47R, D154G | 1.2.2 |
| RBB394 | - | V93V | W47R, D154G | 1.2.2 |
| RBB397 | - | V93V | W47R, D154G | 1.2.2 |
| RBB401 | **E270A** | - | W47R, D154G, S232S | 2.2.1 |
| RBB402 | **E270A** | - | W47R, D154G, S232S | 2.2.1 |
| RBB384 | - | - | W47R, D154G | 4.1.1.3 |
| RBB399 | - | - | W47R, D154G, 1515insG | 4.1.1.3 |
| RBB404 | - | - | W47R, D154G | 4.1.1.3 |
| RBB387 | - | - | W47R, D154G | 4.1.2 |
| RBB392 | - | - | W47R, D154G | 4.1.2 |
| RBB386 | **G152S,** G72G | **P306L** | W47R, D154G | 4.3.3 |
| RBB396 | G72G | **P306L** | W47R, D154G | 4.3.3 |
| RBB403 | - | **P306L** | W47R, D154G | 4.3.3 |
| RBB391 | - | **P306L** | W47R, D154G | 4.8 |
| RBB390 | - | **P306L** | W47R | 4.9 |
| RBB393 | - | **P306L** | W47R | 4.9 |
| RBB400 | - | **P306L** | W47R | 4.9 |

Mutations found in the three methyltransferases (MTases): *mamA*, *hsdM* and *mamB*. In bold, mutations involving amino-acidic changes potentially associated with the loss of function of the MTases, with novel candidates that might impact function of MTases underlined. Cells in red correspond to strains that did not present any of the motifs modified by those MTases methylated.

**Supplementary Figure S1**

**The analytical workflow. (A)** Differential gene expression analysis by clade (between ancient and modern strains). **(B)** eQTL analysis at whole-genome scale, looking for statistical associations between the 9,384 SNPs and 3,987 transcripts in the 22 samples. **(C)** *cis*-eQTL analysis using intragenic or <200 bp upstream SNPs from genes tested for differential transcription. **(D)** *tr*-eQTL analysis looking at the association between transcriptional regulators harbouring potential impairing mutations and differential transcription of genes found within their regulation networks. **(E)** Differential gene expression analysis linked with methylation status (intragenic or in promoter regions).

**Supplementary Figure S2**

**Differential expression of genes non-methylated only in sample with G152S mutation in lineage 4**

Heatmap with the 28 genes differentially expressed among L4 isolates, associated with the lack of methylation in the sample harbouring the mutation G125S in *mamA* (RBB386), constructed with the gene expression distances between rows. Over-expressed genes are coloured in red and under-expressed ones in green. The isolate with none of the CTCCAG motifs methylated is bordered on the left of the white vertical line.

**Supplementary Figure S3**

**Differential expression of genes that non-methylated in Lineage 4 samples**

Heatmap with the 16 genes differentially expressed among Lineage 4 samples associated with the lack of methylation of the different motifs, constructed with the gene expression distances between rows. The 28 genes that were non-methylated only in the strain that contained the G152S mutation are not shown. Over-expressed genes are coloured in red whilst under-expressed ones in green. Bordered cells represent the non-methylated samples for each gene. Bordered in orange are CTCCAG motifs, in yellow are CACGCAG motifs, and in white are GATN_4_RTAC motifs.

**Supplementary Figure S4**

**Comparison of expression levels of genes differentially expressed in lineage 4 clinical isolates with methylated and non-methylated motifs in intergenic regions upstream.**

Boxplots showed the quartiles and median of the log10 of the expression levels for each gene labelled in the x-axis. Red boxplots represent those clinical isolates where the motif found in the upstream intergenic region is not methylated, whilst blue ones represent the isolates where it is methylated. Black points represent the number of samples falling in each of the two groups. When showing a line instead of a boxplot, only one sample is in the group.
